# Supplementary material for: Mineral analysis of complete dog and cat foods in the UK and compliance with European guidelines
Source: Sci Rep. 2017 Dec 7;7:17107. doi: 10.1038/s41598-017-17159-7 (PMC5719410; doi:10.1038/s41598-017-17159-7)
Supplement: Supplementary file 1 — Supplementary Information [file 41598_2017_17159_MOESM1_ESM.pdf]

Title: Mineral analysis of complete dog and cat foods in the UK and compliance with European guidelines

Authors: Davies, M., Jones, L., Alborough, R., Davis, C., Williams, C. and Gardner D.S.

Supplementary Information

Table S1. FEDIAF recommended mineral levels for dogs - Unit per 100 g dry matter (DM)

		Nutritional minimum				Maximum
		Adult MER (kcal/kg$^{0.75}$)		Early Growth (< 14 weeks) & Reproduction	Late Growth (≥ 14 weeks)	
**Mineral**	**Unit**	**95 kcal**	**110kcal**			
Calcium	g	0.58	0.50	1	0.81	Adult: 2.50 (N)<br>Early growth: 1.60 (N)<br>Late growth: 1.80 (N)
Phosphorus	g	0.46	0.40	0.90	0.70	Adult: 1.60 (N)
Ca:P ratio	g	1 : 1				Adult: 2:1 (N)<br>Early growth & Reprod.: 1.6:1 (N)<br>Late growth: 1.8:1* (N)<br>1.6:1** (N)
Potassium	g	0.58	0.50	0.44	0.44	
Sodium	g	0.12	0.10	0.22	0.22	Adult: 1.80 (N)
Chloride	g	0.17	0.15	0.33	0.33	Adult: 2.25 (N)
Magnesium	g	0.08	0.07	0.04	0.04	
Copper	mg	0.83	0.72	1.10	1.10	2.80 (L)
Iodine	mg	0.12	0.11	0.15	0.15	1.10 (L)
Iron	mg	4.17	3.60	8.80	8.80	142.00 (L)
Manganese	mg	0.67	0.58	0.56	0.56	17.00 (L)
Selenium	µg	35.00	30.00	40.00	40.00	56.80 (L)
Zinc	mg	8.34	7.20	10.00	10.00	28.40 (L)

Table S1: Units are given as recommended amount (g, mg or µg as indicated) per 100g of dry matter (DM, assuming 100g = 400 kcal; NRC 2006). *SUL* = safe upper limit MER = metabolic energy requirement; N, nutritional maximum; L, Legal maximum; *, males, **, females. The legal maximum refers to levels of incorporation in additives to the diet, rather than the levels naturally present in the food ingredients.

Table S2: National Research Council (NRC) minimum mineral requirements per kg of diet as fed.

Major element (per kg DM^{-1})	Dogs		Cats	
	Pup (SUL)	Adult (SUL)	Kitten (SUL)	Adult (SUL)
Calcium (g)	8 (18)	2(NS)	5.2 (NS)	1.6 (NS)
Phosphorus (g)	NS (NS)	NS (NS)	4.8 (NS)	1.4 (NS)
Sodium (mg)	NS (NS)	NS (>15)	1240 (>10g)	NS (NS)
Magnesium (mg)	180 (NS)	180 (NS)	160 (NS)	200 (>15)
Potassium (g)	NS (NS)	NS (NS)	2.68 (NS)	NS (NS)
**Trace element (per kg DM^{-1})**				
Zinc (mg)	40 (NS)	NS (NS)	50 (NS)	NS (>600)
Iron (mg)	72 (NS)	NS (NS)	70 (NS)	NS (NS)
Copper (mg)	NS (NS)	NS (NS)	4.5 (NS)	NS (NS)
Manganese (mg)	NS (NS)	NS (NS)	NS (NS)	NS (NS)
Selenium (µg)	210 (NS)	NS (NS)	120 (NS)	NS (NS)

Table S2: Values are given as recommended amount (mg, g or µg as indicated) per kg of dry matter (DM, assuming 1kg=4000 kcal; NRC 2006). *SUL* = safe upper limit NS = minimum requirement not specified.

Table S3: The Association of American Feed Control Officials (AAFCO) complete food guidelines.

Major element (per kg DM^{-1})	Dogs		Cats	
	Pup (max)	Adult (max)	Kitten (max)	Adult (max)
Calcium (%)	1.0 (2.5)	0.6 (2.5)	1.0 (NS)	0.6 (NS)
Phosphorus (%)	0.8 (1.6)	0.5 (1.6)	0.8 (NS)	0.5 (NS)
Ca:P	1:1 (2:1)	1:1 (2:1)	1:1 (2:1)	1:1 (2:1)
Sodium (%)	0.3 (NS)	0.06 (NS)	0.2 (NS)	0.2 (NS)
Magnesium (%)	0.04 (0.3)	0.04 (0.3)	0.08 (NS)	0.04 (NS)
Potassium (%)	0.6 (NS)	0.6 (NS)	0.6 (NS)	0.6 (NS)
**Trace element (per kg DM^{-1})**				
Zinc (mg)	120 (1000)	120 (1000)	75 (2000)	75 (2000)
Iron (mg)	80 (3000)	80 (3000)	80 (NS)	80 (NS)
Copper (mg)	7.3 (250)	7.3 (250)	5.0 (NS)	5.0 (NS)
Manganese (mg)	5.0 (NS)	5.0 (NS)	7.50 (NS)	7.50 (NS)
Selenium (mg)	0.11 (2.0)	0.11 (2.0)	0.10 (NS)	0.10 (NS)

Table S3: Values are given as recommended amount (%, mg, g or µg as indicated) per kg of dry matter (DM). 1% assumed 1g per 100g food. *Max* = nutritional maximum limit. NS = minimum requirement not specified.

Table S4: The Association of American Feed Control Officials (AAFCO) complete food guidelines.

Major element (mg kg DM^{-1})	Complete feeds for pets	
	maximum	exception
Arsenic	2.0	10*
Cadmium	0.5	2**
Mercury	0.1	0.3***
Lead	5	-

Table S4: Values are given maximum level in feed per kg of dry matter (DM). Exceptions are: *, complete feed for pet animals containing fish, other aquatic animals and products derived thereof and/or seaweed meal and feed materials derived from seaweed; **, complete feed for pet animals; ***, compound feed for dogs, cats and fur animals.

Equations used to calculate energy content of complete, wet and dry cat and dog pet foods.

Equation 1: Crude Protein (CP) per DM^{-1} (%):

$$\frac{\underline{CP \ = Protein\ as\ fed\ (\%)}}{}$$
$$(100 - Moisture)/100$$

Equation 2: Nitrogen-free extract (NFE; %)

$$Nfree\ extract = 100 - (Moisture + Fat + Protein + Fibre + Ash)$$

Equation 3: Gross energy (GE) as fed (kcal):

$$GE = (CP\ \times 5.7) + (Fat\ \times 9.4) + ((Fibre + NFE) \times 4.1))$$

Equation 4: Digestible energy (DE) as fed (kcal):

$$DE = \frac{GE\ \times (87.9 - (0.88\ \times Fibre))}{100}$$

Equation 5: Metabolisable energy as fed (kcal):

$$ME = DE - (0.77\ \times CP\ per\ DM)$$

Equation 6: Atwater energy (AT) as fed (kcal):

$$Atwater = (CP\ \times 3.5) + (Fat\ \times 8.5) + (NFE \times 3.5)$$

Equation 7: Atwater energy (AT) kcal per 100g DM^{-1}:

$$Atwater\ DM = (CP \cdot DM^{-1}\ \times 3.5) + (Fat \cdot DM^{-1}\ \times 8.5) + (NFE \cdot DM^{-1}\ \times 3.5)$$

Table S5: Analytical characteristics for mineral analysis of pet foods

Major element (ppm)	Certified Value (Median, ± 15%)	Recovery (Median, %)	LOD	LOQ
Sulphur	7490 (6366,8613)	7131 (95)	7.87	26.2
Calcium	131 (111,150)	127 (97)	0.075	0.25
Phosphorus	11750 (9987,13512)	12024 (102)	0.082	0.27
Sodium	2033 (1728,2337)	1916 (94)	0.079	0.26
Magnesium	620 (527,713)	575 (93)	0.017	0.05
Potassium	10230 (8695,11764)	9713 (95)	0.067	0.22
**Trace element (ppb)**				
Zinc	181 (153,208)	192 (106)	10.17	33.9
Iron	197 (168,227)	4054 (67.5)	0.64	2.16
Copper	275 (233,316)	44.8 (25.7)	0.11	0.36
Manganese	10.4 (8.8,12.0)	10.3 (98)	0.087	0.29
Strontium	0.09 (0.08,0.10)	0.10 (105)	0.08	0.27
Selenium	2.03 (1.72,2.33)	2.15 (106)	0.31	1.06
Chromium	0.05 (0.04,0.06)	0.05 (95)	0.14	0.48
Lead	0.06 (0.05,0.07)	0.05 (89)	0.19	0.64
Vanadium	0.008 (0.006,0.009)	0.009 (109)	0.048	0.16
Molybdenum	3.3 (2.8,3.7)	3.48 (106)	0.19	0.64
Rubidium	35.3 (30.0,40.5)	32.2 (91)	0.093	0.31
Arsenic	0.01 (0.01,0.02)	0.02 (98)	0.33	1.12
Cadmium	0.09 (0.08,0.11)	0.09 (102)	0.062	0.20
Cobalt	0.30 (0.25,0.34)	0.30 (101)	0.019	0.06
Caesium	0.02 (0.01,0.02)	0.02 (105)	0.021	0.072

Table S5. Certified Reference Material was obtained from the National Institutes of Standards and Technology (NIST) using bovine liver 1577c. Elements were measured in duplicate samples of freeze-dried, homogenized and nitric acid-digested material by ICP-MS. Recovery, median value (% recovery) was calculated from an average of 17 independent runs. Limits of detection (LOD) and quantification (LOQ) were calculated from calculating standard deviation (SD) of 10 operational blank samples as LOD = 3.25 × SD (students t statistic, *df*=9, 99% CI) and LOQ = LOD × 2.5.

Figure S1. Guaranteed versus UoN analytical characteristics for mineral analysis of pet foods

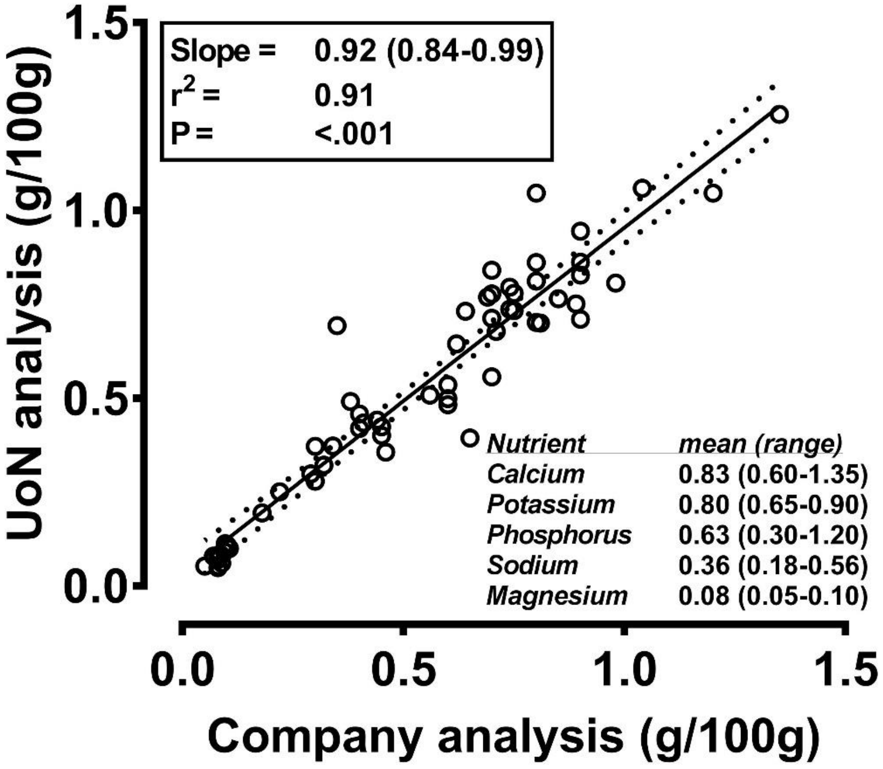

Figure S1. 12 veterinary diets with guaranteed analysis for 5 minerals were compared against values determined by ICP-MS at The University of Nottingham. Solid and dotted lines represent linear regression with 95% C.I. Slope and statistics were determined in Graphpad Prism (v6; Graphpad Software Inc, La Jolla, USA).

Figure S2: Declared ash content vs total mineral composition of wet and dry pet foods.

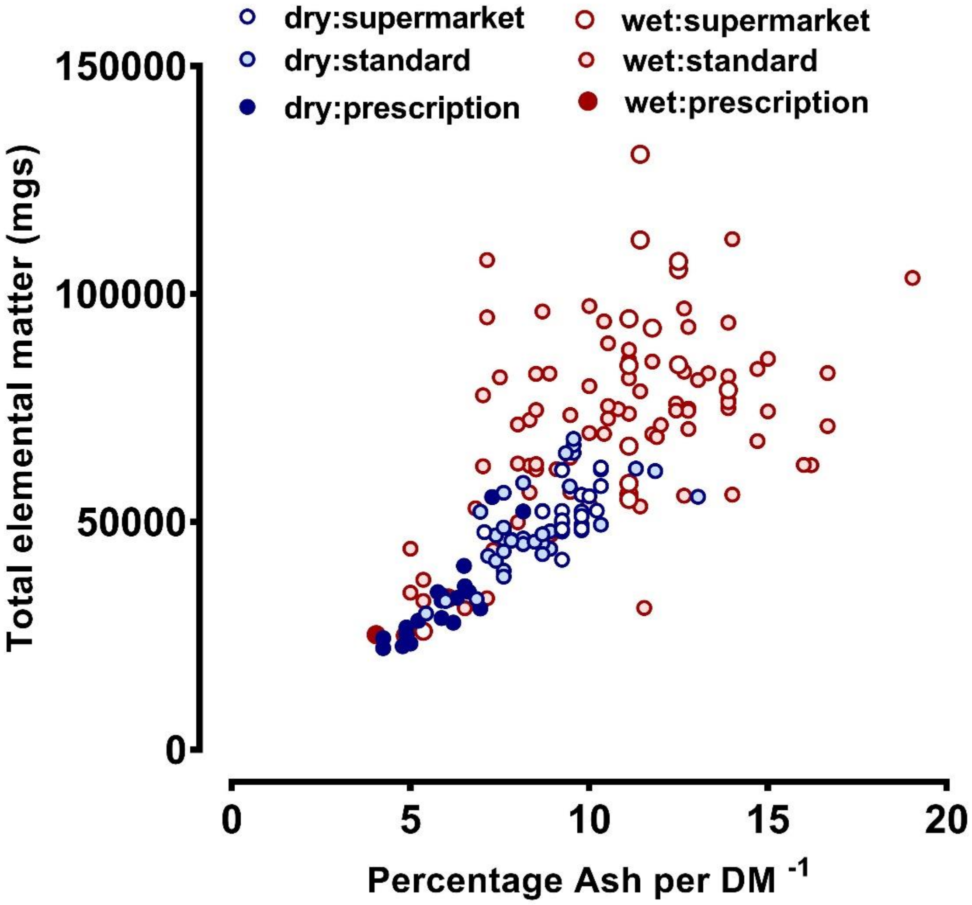

Figure S2: Data are sum of all elements measured by ICP-MS. Each dot represents average of duplicate samples for individual pet foods (wet and dry) designated as 'Supermarket' – labelled as supermarket own brand or 'Prescription' - labelled as a veterinary or therapeutic diet. All other diets were classified as 'Standard'. Ash content was corrected for moisture (i.e. percentage per kg of dry matter (DM)). 1% assumes 1g of minerals per 100g food.

Figure S3: Representation of all foods analysed for compliance with FEDIAF guidelines (DRY FOODS).

SampleID	Company	Mainflavour	FeedQuality	Species	Age	FEDIAF 1: Ca	FEDIAF 2: P	FEDIAF 3: Ca:P	FEDIAF 4: K	FEDIAF 5: Na	FEDIAF 7: Mg	FEDIAF 8: Cu	FEDIAF 10: Fe	FEDIAF 11: Mn	FEDIAF 12: Se	FEDIAF 13: Zn	Total compliant (*n* /11)
S#092		Cereal	Prescription	Cat	Adult												7
S#093		Rice	Prescription	Dog	Adult												8
S#094		Rice	Prescription	Dog	Adult												8
S#097		Cereal	Prescription	Cat	Adult												8
S#142		Cereal	Prescription	Cat	Adult												8
S#143		Chicken	Standard	Dog	Adult												8
S#144		Lamb	Standard	Dog	Adult												8
S#145		Chicken	Prescription	Cat	Adult												9
S#146		Cereal	Prescription	Dog	Adult												9
S#189		Cereal	Prescription	Cat	Senior												9
S#190		Chicken	Prescription	Cat	Adult												9
S#191		Rice	Prescription	Cat	Adult												9
S#192		Fish	Standard	Dog	Puppy												9
S#195		Chicken	Standard	Cat	Adult												9
S#197		Chicken	Standard	Cat	Kitten												9
S#198		Fish	Standard	Cat	Adult												9
S#208		Beef	Supermarket	Dog	Adult												9
S#046		Chicken	Prescription	Cat	Senior												10
S#047		Fish	Standard	Cat	Adult												10
S#048		Cereal	Standard	Dog	Senior												10
S#049		Chicken	Standard	Dog	Adult												10
S#050		Chicken	Standard	Cat	Senior												10
S#096		Fish	Standard	Cat	Adult												10
S#150		Beef	Standard	Dog	Adult												10
S#151		Chicken	Standard	Cat	Senior												10
S#152		Chicken	Standard	Cat	Adult												10
S#153		Chicken	Standard	Cat	Kitten												10
S#154		Fish	Standard	Cat	Adult												10
S#155		Turkey	Standard	Cat	Adult												10
S#156		Chicken	Standard	Cat	Kitten												10
S#157		Chicken	Standard	Cat	Senior												10
S#158		Chicken	Standard	Cat	Adult												10
S#160		Duck	Standard	Cat	Adult												10
S#161		Chicken	Standard	Cat	Adult												10
S#162		Duck	Standard	Cat	Adult												10
S#163		Cereal	Supermarket	Dog	Adult												10
S#170		Chicken	Supermarket	Cat	Senior												10
S#171		Chicken	Supermarket	Cat	Adult												10
S#172		Chicken	Supermarket	Cat	Senior												10
S#173		Fish	Supermarket	Cat	Adult												10
S#174		Chicken	Supermarket	Cat	Adult												10
S#175		Cereal	Supermarket	Cat	Adult												10
S#209		Chicken	Prescription	Cat	Adult												10
S#210		Chicken	Prescription	Cat	Senior												10
S#211		Chicken	Prescription	Cat	Adult												10
S#212		Chicken	Prescription	Cat	Kitten												10
S#095		Chicken	Standard	Cat	Kitten												11
S#149		Fish	Standard	Cat	Adult												11
S#159		Chicken	Standard	Cat	Senior												11
S#164		Chicken	Standard	Cat	Kitten												11
S#165		Turkey	Standard	Cat	Kitten												11
S#166		Turkey	Standard	Cat	Adult												11
S#167		Turkey	Standard	Cat	Adult												11
S#168		Fish	Standard	Cat	Senior												11
S#169		Chicken	Supermarket	Dog	Adult												11
S#176		Chicken	Prescription	Cat	Adult												11
S#177		Cereal	Prescription	Cat	Adult												11
S#178		Cereal	Prescription	Dog	Adult												11
S#179		Fish	Supermarket	Cat	Adult												11
S#180		Duck	Supermarket	Cat	Adult												11
S#181		Fish	Supermarket	Cat	Adult												11
S#182		Fish	Supermarket	Cat	Adult												11
S#183		Duck	Supermarket	Cat	Adult												11
S#184		Fish	Supermarket	Cat	Adult												11
S#185		Chicken	Supermarket	Cat	Kitten												11
S#186		Fish	Supermarket	Cat	Adult												11
S#187		Chicken	Supermarket	Cat	Kitten												11
S#188		Chicken	Prescription	Cat	Adult												11
S#193		Chicken	Prescription	Cat	Adult												11
S#194		Chicken	Prescription	Cat	Adult												11
S#196		Turkey	Standard	Cat	Adult												11
S#199		Duck	Standard	Cat	Adult												11
S#200		Duck	Standard	Cat	Adult												11
S#201		Turkey	Standard	Cat	Senior												11
S#202		Rice	Standard	Dog	Senior												11
S#203		Cereal	Standard	Dog	Adult												11
S#204		Chicken	Standard	Cat	Senior												11
S#205		Chicken	Standard	Cat	Senior												11
S#206		Chicken	Standard	Cat	Adult												11
S#207		Fish	Supermarket	Cat	Adult												11
	Total compliant (*n* /80)					72	70	64	76	80	79	79	80	80	51	78	80

Figure: Each product is represented in an individual row and compliance with European regulation denoted by the averaged value (measured as µg g DM^{-1}) converted to µgs/mgs/g per 100g dry matter (DM) and pictorial represented as a yellow box (BELOW nutritional minimum), red box (ABOVE nutritional maximum) or green box (WITHIN guidelines). Compliance is summated for individual elements (COLUMNS) or products (ROWS).

Figure S4: Representation of all foods analysed for compliance with FEDIAF guidelines (WET FOODS).

SampleID	Company	Mainflavour	FeedQuality	Species	Age	FEDIAF 1: Ca	FEDIAF 2: P	FEDIAF 3: Ca:P	FEDIAF 4: K	FEDIAF 5: Na	FEDIAF 7: Mg	FEDIAF 8: Cu	FEDIAF 10: Fe	FEDIAF 11: Mn	FEDIAF 12: Se	FEDIAF 13: Zn	Total compliant (*n* /11)
S#032		Fish	Standard	Cat	young												5
S#089		Chicken	Standard	Dog	Young												5
S#052		Fish	Standard	Dog	Adult												6
S#062		Lamb	Standard	Dog	Adult												6
S#073		Beef	Standard	Dog	Adult												6
S#077		Chicken	Supermarket	Dog	Adult												6
S#054		Beef	Standard	Dog	Adult												7
S#055		Turkey	Standard	Dog	Adult												7
S#061		Tripe	Standard	Dog	Adult												7
S#069		Beef	Supermarket	Cat	Adult												7
S#083		Version	Standard	Dog	Adult												7
S#086		Fish	Standard	Dog	Adult												7
S#221		Lamb	Standard	Dog	Adult												7
S#222		Beef	Standard	Dog	Adult												7
S#223		Poultry	Standard	Dog	Adult												7
S#232		Fish	Standard	Cat	Adult												7
S#004		Chicken	Standard	Cat	Adult												8
S#005		Turkey	Standard	Cat	Adult												8
S#006		Fish	Standard	Cat	Senior												8
S#026		Chicken	Standard	Cat	Adult												8
S#029		Fish	Standard	Cat	Senior												8
S#042		Lamb	Standard	Dog	Adult												8
S#043		Lamb	Standard	Dog	Adult												8
S#053		Chicken	Standard	Dog	Adult												8
S#058		Lamb	Standard	Dog	Adult												8
S#063		Fish	Standard	Dog	Adult												8
S#068		Unknown	Supermarket	Dog	Adult												8
S#070		Lamb	Standard	Dog	Adult												8
S#074		Fish	Standard	Dog	Adult												8
S#076		Chicken	Standard	Dog	Adult												8
S#082		Tripe	Standard	Dog	Adult												8
S#088		Duck	Standard	Dog	Adult												8
S#225		Turkey	Standard	Dog	Adult												8
S#233		Poultry	Standard	Cat	Adult												8
S#234		Fish	Standard	Cat	Adult												8
S#235		Beef	Standard	Cat	Adult												8
S#001		Beef	Standard	Cat	Adult												9
S#002		Fish	Standard	Cat	Adult												9
S#012		Lamb	Standard	Cat	Young												9
S#014		Turkey	Standard	Cat	Senior												9
S#015		Turkey	Standard	Cat	Adult												9
S#018		Beef	Standard	Cat	Senior												9
S#019		Chicken	Standard	Cat	Adult												9
S#020		Chicken	Standard	Cat	Adult												9
S#023		Chicken	Standard	Cat	Adult												9
S#030		Lamb	Standard	Cat	Adult												9
S#036		Fish	Standard	Cat	Adult												9
S#037		Fish	Standard	Cat	Adult												9
S#044		Beef	Standard	Cat	Senior												9
S#051		Lamb	Standard	Dog	Senior												9
S#064		Duck	Standard	Dog	Adult												9
S#065		Chicken	Standard	Dog	Adult												9
S#066		Chicken	Standard	Dog	Adult												9
S#067		Chicken	Standard	Dog	Adult												9
S#075		Chicken	Standard	Dog	Adult												9
S#080		Fish	Standard	Cat	Adult												9
S#081		Beef	Supermarket	Dog	Adult												9
S#085		Lamb	Standard	Dog	Adult												9
S#214		Poultry	Prescription	Cat	Adult												9
S#219		Fish	Supermarket	Cat	Adult												9
S#220		Poultry	Supermarket	Cat	Adult												9
S#224		Fish	Standard	Dog	Adult												9
S#226		Fish	Standard	Dog	Adult												9
S#227		Poultry	Standard	Dog	Adult												9
S#229		Fish	Standard	Cat	Adult												9
S#230		Poultry	Standard	Cat	Adult												9
S#003		Chicken	Standard	Cat	Adult												10
S#007		Rabbit	Standard	Dog	Adult												10
S#008		Beef	Standard	Cat	Adult												10
S#009		Rabbit	Standard	Cat	adult												10
S#010		Turkey	Standard	Cat	Adult												10
S#011		Chicken	Standard	Cat	Adult												10
S#013		Lamb	Standard	Cat	Senior												10
S#016		Turkey	Standard	Cat	young												10
S#017		Gravy	Standard	Cat	Senior												10
S#028		Fish	Standard	Cat	Adult												10
S#031		Fish	Standard	Cat	Adult												10
S#041		Beef	Standard	Dog	Adult												10
S#045		Fish	Standard	Cat	Adult												10
S#057		Chicken	Standard	Cat	Adult												10
S#059		Lamb	Standard	Dog	Adult												10
S#060		Venison	Standard	Dog	Adult												10
S#071		Fish	Standard	Cat	Adult												10
S#079		Chicken	Standard	Dog	Adult												10
S#090		Chicken	Standard	Dog	Adult												10
S#213		Poultry	Prescription	Dog	Adult												10
S#215		Cereal	Prescription	Dog	Adult												10
S#216		Fish	Supermarket	Cat	Adult												10
S#217		Fish	Supermarket	Cat	Adult												10
S#218		Fish	Supermarket	Cat	Adult												10
S#231		Fish	Standard	Cat	Adult												10
S#034		Beef	Standard	Cat	Adult												11
S#072		Beef	Standard	Dog	Adult												11
S#078		Fish	Standard	Dog	Adult												11
S#084		Chicken	Standard	Dog	Adult												11
S#087		Rabbit	Standard	Dog	Senior												11
S#091		Duck	Standard	Dog	Adult												11
Total compliant (*n* /97)						76	72	69	96	94	96	66	87	91	18	85	97

Figure: Each product is represented in an individual row and compliance with European regulation denoted by the averaged value (measured as µg g DM^{-1}) converted to µgs/mgs/g per 100g dry matter (DM) and pictorial represented as a yellow box (BELOW nutritional minimum), red box (ABOVE nutritional maximum) or green box (WITHIN guidelines). Compliance is summated for individual elements (COLUMNS) or products (ROWS).
